# Werner Syndrome Protein and DNA Replication

**DOI:** 10.3390/ijms19113442

**Published:** 2018-11-02

**Authors:** Shibani Mukherjee, Debapriya Sinha, Souparno Bhattacharya, Kalayarasan Srinivasan, Salim Abdisalaam, Aroumougame Asaithamby

**Affiliations:** Division of Molecular Radiation Biology, Department of Radiation Oncology, University of Texas Southwestern Medical Center, Dallas, TX 75390, USA; Shibani.Mukherjee@utsouthwestern.edu (S.M.); Debapriya.Sinha@utsouthwestern.edu (D.S.); Souparno.Bhattacharya@utsouthwestern.edu (S.B.); Kalayarasan.Srinivasan@utsouthwestern.edu (K.S.); Salim.Abdisalaam@utsouthwestern.edu (S.A.)

**Keywords:** cancer, DNA double-strand repair, premature aging, post-translational modification, protein stability, replication stress, Werner Syndrome, Werner Syndrome Protein

## Abstract

Werner Syndrome (WS) is an autosomal recessive disorder characterized by the premature development of aging features. Individuals with WS also have a greater predisposition to rare cancers that are mesenchymal in origin. Werner Syndrome Protein (WRN), the protein mutated in WS, is unique among RecQ family proteins in that it possesses exonuclease and 3′ to 5′ helicase activities. WRN forms dynamic sub-complexes with different factors involved in DNA replication, recombination and repair. WRN binding partners either facilitate its DNA metabolic activities or utilize it to execute their specific functions. Furthermore, WRN is phosphorylated by multiple kinases, including Ataxia telangiectasia mutated, Ataxia telangiectasia and Rad3 related, c-Abl, Cyclin-dependent kinase 1 and DNA-dependent protein kinase catalytic subunit, in response to genotoxic stress. These post-translational modifications are critical for WRN to function properly in DNA repair, replication and recombination. Accumulating evidence suggests that WRN plays a crucial role in one or more genome stability maintenance pathways, through which it suppresses cancer and premature aging. Among its many functions, WRN helps in replication fork progression, facilitates the repair of stalled replication forks and DNA double-strand breaks associated with replication forks, and blocks nuclease-mediated excessive processing of replication forks. In this review, we specifically focus on human WRN’s contribution to replication fork processing for maintaining genome stability and suppressing premature aging. Understanding WRN’s molecular role in timely and faithful DNA replication will further advance our understanding of the pathophysiology of WS.

## 1. Introduction

Werner Syndrome (WS) is an autosomal recessive genetic disorder that causes symptoms of premature aging and is accompanied by a higher risk of cancer [1,2,3]. Individuals with WS show a greater predisposition to diseases usually observed in older age, such as arteriosclerosis, cataracts, osteoporosis, and type II diabetes mellitus [4,5,6]. In addition, individuals with WS are more susceptible to rare cancers that are mesenchymal in origin [1,2]. Myocardial infarction and cancer are the most common causes of death among patients with WS [2]. Primary cells derived from these patients exhibit elevated levels of chromosomal translocations, inversions, and deletions of large segments of DNA, and they have a high spontaneous mutation rate [7,8]. Additionally, WS fibroblasts have a markedly shorter replicative life span than age-matched controls in culture [4,9]. Most WS cases have been linked to mutations in a single gene, the Werner syndrome gene (*WRN*), which is located on chromosome 8 [10].

**Figure 1:**
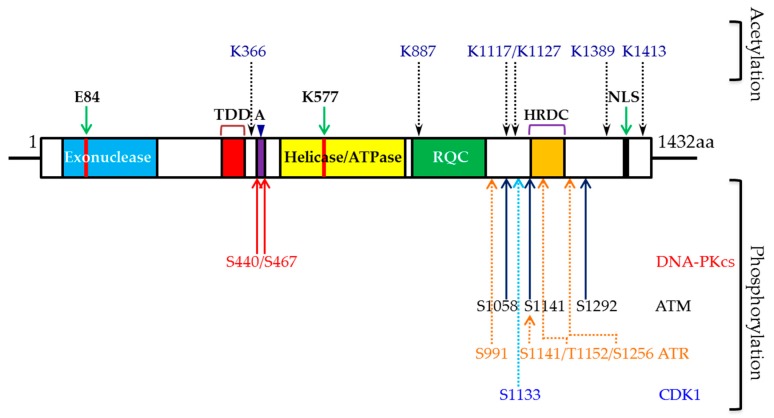
Schematic showing different functional domains, exonuclease (E84), helicase (K577) active sites, and DNA-PKcs (S440 and S467), ATM (S1058, S1141 and S1292), ATR (S991, S1411, T1152 and S1256) and CDK1 (S1133) phosphorylation, and acetylation (K366, K887, K1117, K1127, K1389 and K1413) sites in WRN. TDD-Trimerization (oligomerization/multimerization) domain (250–333aa); A-acidic repeats (2X27; 424–477 aa); RQC-RecQ C-terminal (749–899 aa); NLS-nuclear localization signal; aa-amino acid; black dotted lines denote acetylation events; solid red arrows indicate DNA-PKcs-mediated phosphorylation sites; solid dark blue lines represent ATM-mediated phosphorylation events; dotted orange arrows represent ATR-dependent phosphorylation sites; light blue dotted line represents CDK1-dependent phosphorylation site.

WRN, the protein defective in WS, belongs to the RecQ helicase family. The human genome contains five RecQ genes: RecQ1, Bloom syndrome protein (BLM), WRN, RecQ4, and RecQ5. WRN is a 1432 amino acid-long multifunctional protein that comprises four distinct functional domains (Figure 1). WRN has an exonuclease (E84) domain (38–236 aa) and a WRN-WRN interaction (multimerization or oligomerization) domain (251–333 aa) in the N-terminal region. It has adenosine triphosphatase (ATPase), helicase (K577) (558–724 aa), and RecQ C-terminal (RQC) (749–899 aa) domains in the middle region and a helicase-and-ribonuclease D-C-terminal (HRDC) domain (940–1432 aa) in the C-terminal region. Though the crystal structure for full-length WRN is not available yet, crystal structures of the exonuclease and HRDC domains have been solved. The crystal structure of the exonuclease domain (1–333 aa) at 2.0 angstrom resolution showed a ring of six WRN exonuclease domains, the perfect size to slip around a DNA helix, with their binding and catalytic sites oriented inward toward the encircled DNA [11]. This study further revealed that WRN’s exonuclease domain possesses Mg^2+^ and Mn^2+^ binding sites, which help modulate WRN’s exonuclease activities [11]. Additionally, full-length WRN forms a trimer [12], and the WRN exonuclease construct (1–333 aa) forms a trimer when purified by gel filtration analysis and homohexamers upon interaction with DNA or with Proliferating cell nuclear antigen (PCNA), as examined by atomic force microscope [13,14]. Subsequently, Perry et al. (2010) identified the 250–333 amino acids as being not only responsible for WRN’s homomultimerization, but also critical for its exonuclease processivity [15]. The HRDC domain’s crystal structure revealed that this domain exists as a monomer in solution and has weak DNA binding ability in vitro [16]. However, the HRDC domain is known to interact with many different proteins, which suggests that WRN’s DNA binding specificity is dictated by another domain. Thus, structural analyses of N- and C-terminal domains have provided a wealth of information about WRN’s exonuclease activities and its ability to act on different DNA structures.

WRN exonuclease functions on a variety of structured DNA substrates, including bubbles, stem-loops, forks, and Holliday junctions, as well as RNA-DNA duplexes, which suggests that WRN may have roles in DNA replication, recombination, and repair [17,18]. WRN’s 3′ to 5′ DNA helicase activity [19] may coordinate with its exonuclease activity, as both show similar substrate specificity. WRN also performs non-enzymatic functions during DNA replication and repair [20,21,22], though the regulation of these activities is poorly understood.

WRN plays roles in many biological processes, as it forms dynamic sub-complexes with factors involved in those processes. WRN directly binds to Nijmegen breakage syndrome protein’s (NBS1) forkhead-associated (FHA) domain in response to DNA double-strand breaks (DSBs). This interaction is important for the post-translational modification of WRN [23]. WRN also interacts with Meiotic recombination 11 homolog A (MRE11) nuclease via NBS1 [24]; MRE11 promotes WRN helicase activity, but WRN does not modulate MRE11’s nuclease activities [24]. WRN interacts with RAD51, but this interaction does not affect WRN’s nuclease activities [25]. WRN physically interacts with Xeroderma pigmentosum complementation group G (XPG) protein, a DNA endonuclease; this interaction is critical for stimulating WRN’s helicase activity [26]. WRN not only directly interacts with Replication protein A (RPA), but also displaces it from the replication forks [27]. WRN interacts with telomeric repeat binding factor 2 (TRF2), which helps WRN’s exonuclease activity to process telomeric repeat DNA [28]. BLM and RecQ5L helicases functionally interact with WRN to modulate its exonuclease and helicase activities, respectively [29]. WRN also interacts with ATR [25] and DNA-dependent protein kinase catalytic subunit (DNA-PK_CS_) [30,31]. Importantly, mutations in most of these genes lead to disorders that predispose to cancer. Yet, the contributions of WRN and its partnering factors to maintaining genome stability and suppressing premature aging phenotypes in response to replication stress are not fully understood.

## 2. WRN in DNA Replication

DNA replication is an intricate process that is monitored closely by a plethora of proteins acting synchronously to create two identical daughter DNA strands from one parental DNA strand while ensuring maximum fidelity. However, a chance for error always remains, which could eventually change the fate of the daughter cells. The various checkpoints in the cell cycle exist to ensure the accurate segregation of DNA to the daughter cells. Numerous studies characterizing WRN have shown that it facilitates replication fork progression, helps restart stalled DNA replication [32,33,34], and protects replication forks [22]. In most cases, either post-translationally modified WRN cooperates with other replication fork processing factors, or its nuclease activities are modulated by its interacting partner and by post-translational modifications under replication stress conditions.

### 2.1. Role of WRN in Replication Fork Progression

WRN has been implicated in replication fork progression and efficient restart of DNA replication under normal physiological and genotoxic agent-induced replication stress conditions [22,32,33,34,35]. An elegant study by Rodriguez-Lopez et al. (2002), using a single-molecule DNA fiber technique, showed that WRN is important in the elongation stage of DNA replication [35]. In the absence of WRN, cells fail to maintain bidirectional DNA replication, resulting in asymmetrical bidirectional forks. Based on these observations, they proposed that the WRN helicase is involved either in preventing the collapse of stalled replication forks or in resolving intermediates present at collapsed forks. A study by Sidorova et al. (2008) found that WRN affects replication fork progression after methyl methanesulfonate (MMS)-induced replication fork damage [33]. Another study by Su et al. (2014) found defects in replication fork progression in response to collapsed replication forks [22]. Collectively, these findings suggest that WRN facilitates the progression of stalled or collapsed replication forks generated under normal physiological conditions and under genotoxic agents induced replication stress conditions.

### 2.2. Role of WRN in Replication Fork Arrest Recovery

WRN function has been strongly implicated in the recovery of arrested replication forks [36,37]. Most of these functions of WRN are carried out in concert with its interacting partners with known roles in DNA replication and its post-translational modifications. WRN interacts with replication checkpoint factors—specifically, the RAD9-RAD1-hydroxyurea-sensitive 1 (HUS1; 9.1.1) complex [38]—to prevent DSBs from forming at stalled replication forks. A study by Pichierri et al. (2012) found that the RAD1 subunit of the 9.1.1 complex binds to WRN’s N-terminal region, which contributes to WRN’s re-localization to nuclear foci and phosphorylation in response to replication fork stalling. They also showed that WRN affects the ATR signaling pathway, promoting checkpoint activation in response to stalled replication forks, and is phosphorylated by ATR in a replication checkpoint mediated protein (TopBP1)-dependent manner, upon replication fork arrest [38]. These coordinated activities of WRN-9.1.1-ATR-TopBP1 are critical for the stability of fragile sites [38]. Replication stress triggers co-localization of WRN with RPA at nuclear foci in a manner that depends on WRN phosphorylation by ATR [34]. Cells expressing an ATR-unphosphorylatable mutant of WRN behave like WRN-deficient cells; stalled replication forks collapse, leading to DSB formation [34]. The accumulation of replication-associated DSBs in WRN-deficient cells depends on structure-specific endonuclease MMS and ultraviolet-sensitive 81 (MUS81) activity [39]. Replication stress leads not only to DSB accumulation but also to increased expression of common fragile sites in WRN-deficient cells [40]. Thus, ATR/ATM-mediated WRN phosphorylation and WRN’s interacting partners are crucial for replication fork arrest recovery.

### 2.3. Role of WRN in Replication Fork Protection

**Figure 2:**
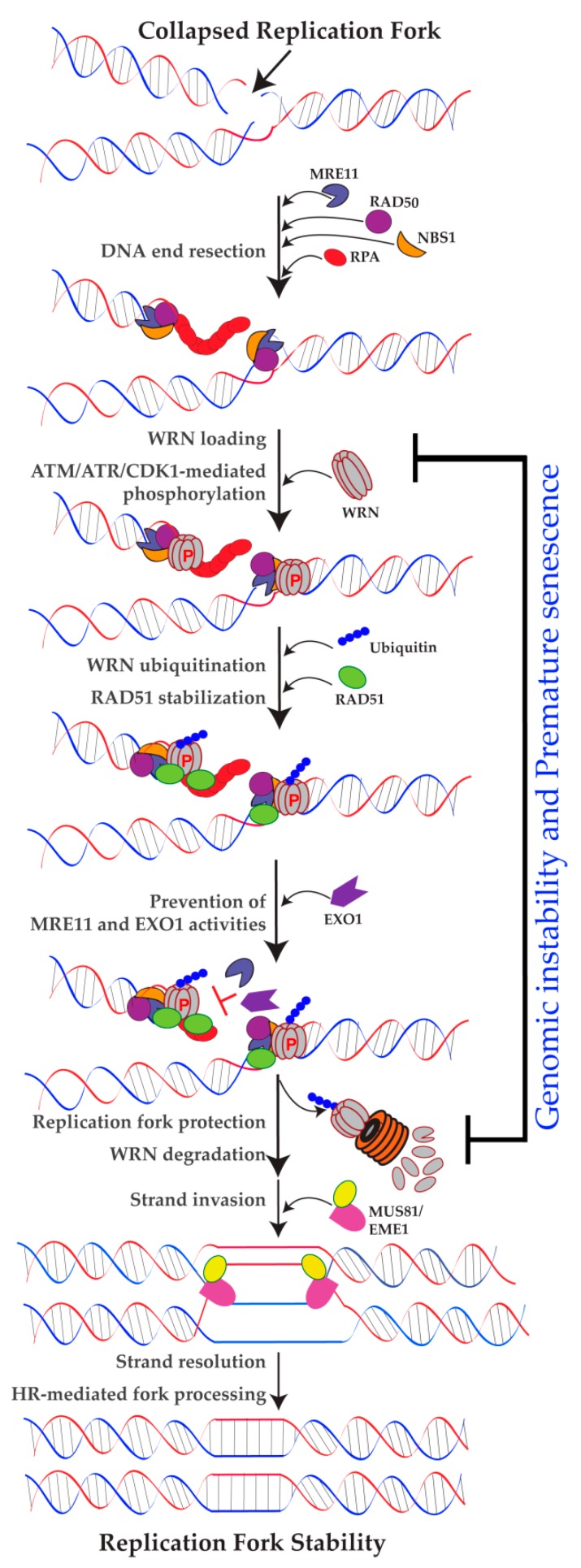
Diagram depicting the mechanism of WRN-mediated replication fork stabilization. WRN is recruited to the sites of collapsed replication forks and is phosphorylated at multiple Ser/Thr sites by ATM, ATR and CDK1 kinases. WRN binding to perturbed replication forks not only stabilizes RAD51 but also prevents excessive nuclease activities of MRE11 and/or EXO1. Eventually, WRN is degraded by the ubiquitin-dependent proteasomal pathway, resulting in the protection of newly replicated genome, chromosome stability and suppression of premature senescence. In the absence of WRN, replication forks will be degraded by MRE11 and/or EXO1, and that will lead to genomic instability and premature senescence. HR—homologous recombination; EXO1—exonuclease 1; RPA—replication protein A; EME1-essential meiotic structure-specific endonuclease 1; red P—phosphorylation events; ⏊-represents blocking of nuclease activities.

In addition to replication fork processing and recovery functions, recent findings strongly suggest roles for WRN in protecting replication forks (Figure 2). An elegant study by Su et al. (2014) reported a non-enzymatic role for WRN in stabilizing newly replicated DNA strands at collapsed replication forks [22]. They found that WRN blocks excessive processing of newly replicated genomic DNA by MRE11 in response to collapsed replication forks. This is an interesting study, because most of the work on WRN focuses on its nuclease activities in various DNA metabolic pathways, but this study identified the physical presence of WRN at the sites of collapsed replication forks as the key for RAD51 stabilization, which blocks MRE11 activities on the newly replicated genome. Another study by Iannascoli et al. (2015) showed that the exonuclease activity of WRN protects MRE11- and Exonuclease 1 (EXO1)-mediated degradation of nascent DNA strands at regressed forks in the absence of significant numbers of DSBs [41]. Additionally, Kehrli et al. (2016) found that WRN not only interacts with Class I Histone Deacetylase (HDAC1), but also co-localizes with HDAC1 on newly replicated DNA. This interaction helps to protect replication forks upon hydroxyurea-induced replication fork arrest [42]. Thus, in addition to roles in replication fork progression and efficient restart, WRN is also involved in maintaining nascent DNA strands by at least three distinct mechanisms in response to replication stress. However, the molecular mechanism by which cells utilize WRN and its exonuclease activity to protect nascent DNA strands in response to replication stress is not clear.

## 3. WRN and Phosphorylation

Phosphorylation is one of the most common post-translational modifications in proteins that occurs in response to genotoxic stress. WRN has been shown to be phosphorylated at serine/threonine and tyrosine in vivo when cells are exposed to different DNA damaging agents [43]. WRN is phosphorylated by several kinases, including ATM, ATR [34,44], DNA-PK_CS_ [30,31,45], c-Abl [46], and CDK1 [47] (Figure 1). WRN phosphorylation may affect its enzymatic activities, protein-protein interactions, stability, and sub-nuclear redistribution. These phosphorylation events in WRN are necessary for maintaining genome stability and suppressing premature aging.

### 3.1. Role of DNA-PK_CS_-Mediated WRN Phosphorylation

WRN is phosphorylated by DNA-PK_CS_ both in vitro and in vivo. A first study by Yannone et al. (2001) demonstrated that WRN associates with the catalytic subunit of DNA-PK (DNA-PK_CS_) and requires KU70/80 to form a stable WRN·DNA-PK·DNA complex in vitro [30]. The association of WRN with DNA-PK_CS_ inhibits WRN’s exonuclease and helicase activities, but adding KU70/80 to form the WRN·DNA-PK·DNA complex activates exonuclease and helicase activities in vitro. Moreover, DNA-PK_CS_–dependent WRN phosphorylation is critical for processing ionizing radiation-induced DSBs [30]. A second report by Karmakar et al. (2002) revealed that WRN interacts with DNA-PK_CS_, and KU70/80 mediates this interaction [31]. Similar to Yannone et al., (2001) they also found that DNA-PK_CS_ phosphorylates WRN both in vitro and in vivo. They also showed that, in contrast to Yannone et al.’s findings, WRN phosphorylation by DNA-PK_CS_ inhibits WRN’s exonuclease activity in the presence of KU70/80, whereas WRN dephosphorylation enhances its exonuclease and helicase activities. A third study identified S440 and S467 in WRN as major phosphorylation sites mediated by DNA-PK [45]. Additionally, the phosphorylation of S440 and S467 in WRN is important for re-localizing WRN to nucleoli, which is required for efficient DSB repair. Thus, WRN is a target of DNA-PK phosphorylation, and its catalytic activities and re-localization are regulated by phosphorylation [30,31,45]. However, no study has addressed whether DNA-PK_CS_-mediated WRN phosphorylation could also occur in response to replication stress.

### 3.2. Role of ATR-Mediated WRN Phosphorylation

In response to replication stress, ATR, one of the members of the phosphatidylinositol 3-kinase-related kinase (PIKK) family, phosphorylates WRN. Ammazzalorso et al. (2010) used anti-phospho Serine-Glutamine (SQ) / Threonine-Glutamine (TQ) antibodies to analyze random SQ/TQ mutations in WRN and found that S991, T1152, and S1256 residues are substrates of ATR kinase [34]. By mutating multiple amino acid residues simultaneously and extrapolating results from multi-site mutants, they found that WRN phosphorylation by ATR prevents the formation of DSBs at stalled replication forks by regulating WRN’s sub-nuclear re-localization and interaction with RPA [34]. Another study by Su et al. (2016), using mass-spectrometry followed by phospho-specific antibodies that recognize the phosphorylated S1141 antibody, found that ATR phosphorylates the S1141 residue in response to replication stress in vivo, but only during active DNA replication [48]. They also found that WRN, RPA2, and RAD51 may be recruited to the sites of perturbed DNA replication without WRN phosphorylation at S1141, but S1141 phosphorylation in vivo helps dissociate RAD51 and WRN from the damaged DNA ends. Thus, ATR-mediated WRN S1141 phosphorylation is important for suppressing chromosome instability in response to collapsed replication forks. Collectively, these studies show that WRN is a substrate of ATR kinase and that WRN phosphorylation by ATR is critical for suppressing genome instability following replication stress induced by genotoxic agents.

### 3.3. Role of ATM-Mediated WRN Phosphorylation

WRN also serves as a substrate for ATM. An in vitro kinase assay-based peptide screening method identified S1141 and S1292 in WRN as ATM phosphorylation sites [49]. Subsequently, Ammazzalorso et al. (2010) found that ATM phosphorylates WRN specifically at S1058, S1141, and S1292 in response to replication stress [34]. Furthermore, using multi-ATM phosphorylation site WRN mutants, the authors deduced that ATM phosphorylation promoted homologous recombination (HR) repair of collapsed forks by influencing RAD51’s ability to form nuclear foci after cells were exposed to hydroxyurea [34]. However, additional experiments are needed to define how ATM-dependent phosphorylation of WRN modulates RAD51 functions at perturbed replication forks.

### 3.4. Role of CDK1-Mediated WRN Phosphorylation

WRN is phosphorylated at S1133 by cyclin-dependent kinase 1 (CDK1) [47]. WRN phosphorylation by CDK1 has multiple consequences: 1. promotion of the DNA replication helicase/nuclease 2 (DNA2)-dependent long-range step in end resection of replication-associated DSBs; 2. dynamic interaction of MRE11 with replication forks; 3. facilitation of HR; 4. replication fork recovery; and 5. genome stability maintenance. It will be interesting to further understand how CDK1-mediated WRN phosphorylation modulates other post-translational modifications in WRN.

## 4. WRN Stability and Degradation

Regulated protein degradation rapidly and irreversibly turns off a protein’s function. This process is critical, since maintaining genomic integrity and cellular homeostasis requires that cells eliminate various proteins related to genome curation and stability, including DNA damage response signaling factors, properly and at the right time [50]. WRN undergoes proteasome-mediated degradation in response to genotoxic stress, and this destabilization of WRN is critical for maintaining genome stability and suppressing premature aging.

### 4.1. Role of WRN Ubiquitination

Results from three independent research groups have identified ubiquitin-dependent destabilization of WRN in response to replication stress [23,48,51]. The first study, by Kobayashi et al. (2010), found that ATM/NBS1-dependent WRN phosphorylation facilitates ubiquitin-dependent degradation of WRN in response to replication stress [23]. The second study, by Su et al. (2016), showed that ATR-mediated WRN phosphorylation influences WRN destabilization and genome stability maintenance. According to Su et al. (2016), ATR-mediated WRN phosphorylation at S1141 leads to proteasome-mediated degradation of WRN following replication stress induced by camptothecin (CPT). Their study used enhanced green fluorescent protein-tagged wild-type, phospho-mutant (S1141A), and phospho-mimetic (S1141D) WRN in combination with fluorescence redistribution after photobleaching (FRAP) technique, and it revealed that ATR-mediated S1141 phosphorylation is critical for the reversible interaction of WRN with collapsed replication forks [48]. Similarly, a third study, by Shamanna et al. (2016), found that replication stress induced by CPT triggered WRN degradation by a ubiquitin-mediated proteasome pathway [51]. Taken together, these reports indicate that WRN is ubiquitinated in response to replication stress, which impacts its interaction with binding partners and its stability. However, neither ubiquitination sites nor ubiquitin ligases that mediate replication stress-dependent WRN ubiquitination have been identified.

ATM- and ATR-dependent WRN phosphorylation is important for proper cellular recovery from replication stress, maintaining genome stability, and avoiding premature senescence. Phosphorylation also redirects WRN to ubiquitin-mediated degradation. However, it is unclear whether WRN degradation takes place after DNA metabolic activities have completed or it facilitates subsequent steps involved in cell recovery from genotoxic stress. Furthermore, WRN phosphorylation facilitates WRN’s dynamic interaction with perturbed replication forks, and in the absence of phosphorylation, WRN tightly interacts with replication-associated DSBs, culminating in chromosome instability [48]. Therefore, it is possible that phosphorylation facilitates WRN’s initial role in DNA processing and that subsequent ubiquitination-mediated proteasomal degradation helps recruit additional factors to the replication forks for faithful replication fork processing. However, further experiments are needed to verify these speculations.

### 4.2. Role of WRN Acetylation

In addition to being phosphorylated and ubiquitinated, WRN is also acetylated in response to replication stress. Li et al. (2010) identified six lysine acetylation sites (K366, K887, K1117, K1127, K1389 and K1413) in WRN, and these acetylation events were mediated by cyclic adenosine monophosphate (cAMP) response element binding (CREB)-binding protein (CBP) and p300 acetyltransferases after cellular exposure to the DNA cross-linking agent mitomycin c (MMC). Furthermore, deacetylase Sirtuin 1 reversed the effects of WRN acetylation [52]. According to Lozada et al. (2014), endogenous WRN is mildly acetylated under normal physiological conditions, and its acetylation level increases in response to stalled replication forks [53]. WRN acetylation in response to replication stress serves multiple functions: 1. it translocates WRN from nucleoplasm to nucleoli; 2. it directs WRN’s binding affinity to four-stranded replication fork structures; 3. it modulates WRN’s exonuclease and helicase activities; and 4. it redirects WRN to the ubiquitin-mediated proteasomal degradation pathway. Though the impact of acetylation on WRN’s functions is similar to that of phosphorylation induced by replication stress, it remains unknown how WRN acetylation influences its phosphorylation, and vice versa, to maintain genome stability or suppress premature aging phenotypes.

## 5. WRN and DSB Repair Pathway Choice

DNA repair is an essential and spontaneous phenomenon that occurs in cells in response to genomic damage induced by various endogenous and exogenous agents. Repairing damaged genome is imperative to avoid complications like mutations and replication stress. The different pathways that the cell adopts to repair DNA lesions are base excision repair (BER), nucleotide excision repair (NER), mis-match repair (MMR), and DSB repair. DSBs are repaired by two major pathways: non-homologous end-joining (NHEJ) and HR. These two pathways differ in the templates they use and have marked differences in their ultimate repair fidelity. NHEJ processes the broken DNA ends and later ligates them, while HR uses an undamaged sister chromatid as a template for repair and, thus, is more accurate than NHEJ. The two pathways’ specific requirements indicate that the choice between them may depend on which stage the cell is in the cell cycle. Numerous studies have established that HR is most active in mid-S (synthetic) to early G2 (second growth) phases, while NHEJ, which can be active in all four phases of the cell cycle, is mostly predominant in the G1 (first growth) and G2/M (mitotic) phases [54,55]. Biochemical and cell biological evidence suggests a potential role for WRN in NHEJ (both c- and alt-NHEJ) and HR, owing to the 3′ to 5′ directionality of the helicase and exonuclease, the hypersensitivity of WS cells to certain genotoxic agents, and WRN’s interaction with proteins involved in these DNA repair pathways [22,48].

### 5.1. Role of WRN in Classical NHEJ

**Figure 3:**
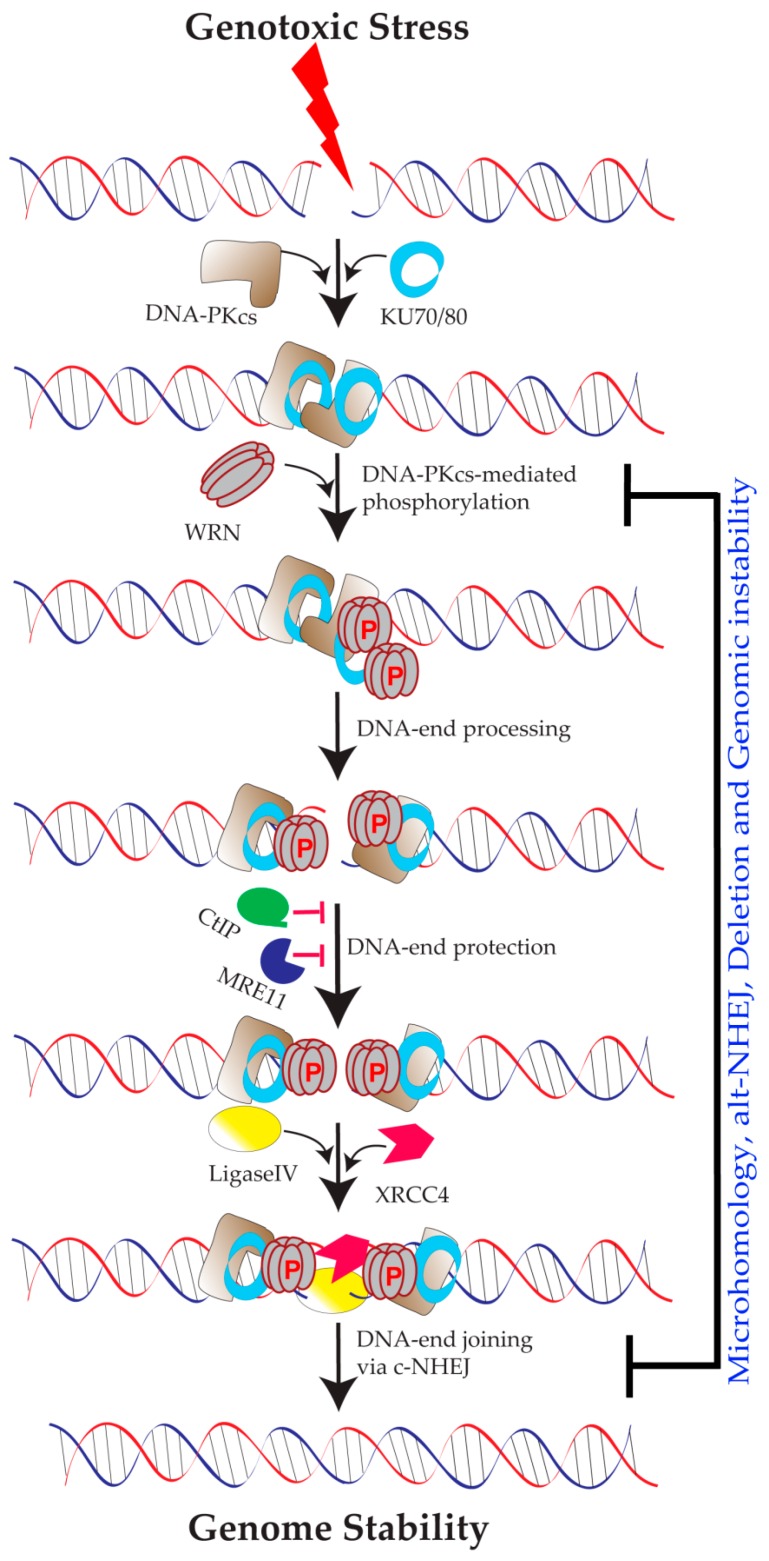
Schematic showing the involvement of WRN in classical non-homologous end-joining (c-NHEJ) pathway-mediated DNA double-strand break (DSB) repair in response to both endogenous and exogenous genotoxic stress. DNA-PK (DNA-PK_CS_+KU70/80) complex not only recruits WRN to DSB sites but also phosphorylates at multiple amino acid residues in WRN. In addition, physical binding of WRN to damaged DNA prevents excessive enzymatic activities of MRE11 and CtIP. These events are necessary for preventing genomic DNA deletions, microhomology-mediated DSB repair, and alt-NHEJ-mediated DSB repair. DNA-PK_CS_—DNA-dependent protein kinase catalytic subunit; NHEJ—non-homologous end joining; P—phosphorylation events; XRCC4-X-ray repair cross complementing 4; CtIP-C-terminal binding protein 1 (CtBP1) interacting protein; rose red ⏊-represents blocking of nuclease activities; red lightening shape arrow denotes genotoxic stress.

The NHEJ pathway has two sub-pathways [56,57]: 1. Classical NHEJ (c-NHEJ) and 2. Alternative NHEJ (alt-NHEJ). WRN interacts not only with major c-NHEJ factors, such as KU 70/80 [58] and DNA-PK_CS_ [30,31], but also a substrate for DNA-PK_CS_ [30,31] (Figure 3). Because DNA-PK and WRN assemble at DNA ends, DNA-PK_CS_ can phosphorylate WRN, and thus regulate WRN’s enzymatic activity and facilitate DNA end processing before ligation [30,31]. It has been shown that WRN deficiency results in the excessive degradation of non-homologous DNA ends during NHEJ pathway repair of exogenously transfected linear plasmid DNA substrate [59]. Cellular end-joining assays using linearized reporter plasmids showed that WRN’s exonuclease and helicase activities influence DNA end-joining [20]. Cellular assays that distinguish microhomology-mediated DSB repair from unmediated repair of linear plasmids demonstrated that WS cells display increased levels of microhomology-mediated DSB repair; introducing WT WRN, but not the exonuclease- or helicase-mutant WRN, in WS cells completely rescued the WS phenotype [11]. Despite physical and functional interactions with c-NHEJ pathway factors, the exact role of WRN in c-NHEJ is not clear.

### 5.2. Role of WRN in Alternative NHEJ Pathway

Cells utilize alt-NHEJ as a back-up DSB repair pathway when the c-NHEJ pathway is defective. Paradoxically, alt-NHEJ has both beneficial and harmful outcomes. For example, alt-NHEJ plays a beneficial role during class switch recombination, an essential process that generates antibody isotopes [60], but DSB repair by the alt-NHEJ pathway also results in genome instability [61,62]. A recent study showed that WRN regulates the pathway choice between c- and alt-NHEJ during DSB repair [63,64]. According to this study, WRN is recruited to the sites of DSBs, in a nuclease activities-dependent manner, which prevents DNA end-resection by blocking MRE11’s and CtIP’s access to DSBs. Thus, the physical presence of WRN at the sites of DSBs blocks MRE11-CtIP-dependent DNA end-resection, thus promoting c-NHEJ via its helicase and exonuclease activities. However, if WRN is absent, MRE11 and CtIP are recruited to the DSBs, which leads to DNA end-resection and culminates in alt-NHEJ. Ultimately, WRN-mediated c-NHEJ protects DSBs from MRE11/CtIP-mediated resection, which prevents the deletion of large genomic regions and telomere fusions. However, further research is needed to understand how WRN-proficient S/G2 phase cells allow DNA end-resection to proceed during HR-mediated replication fork processing.

### 5.3. Role of WRN in HR

HR repair (HRR) is a multi-step process that requires the coordinated activities of different proteins. HRR involves three major steps (Figure 2). First, DNA end-resection generates single-strand DNA overhangs, resulting in the recruitment of RPA, RAD51 and their interacting partners. Then, strand invasion results in the formation of D-loops and Holiday junctions (HJ). Finally, HJ resolution results in processed duplex DNA. Since HRR uses the sister chromatid to repair the complementary sequence, this mode of DNA repair is well-suited to repairing a multitude of DNA lesions, including stalled or collapsed replication forks, that may be triggered during normal physiological conditions or by various genotoxic agents.

Multiple studies have shown that DNA damaging agents that cause replication fork stalling and treatments with replication inhibitors have deleterious effects on WS cells [65,66,67,68,69]. For example, WRN-deficient primary fibroblasts are hypersensitive to 4-nitroquinoline-1-oxide (4NQO) [70,71], topoisomerase I inhibitors (camptothecin) and DNA cross-linking agents [72]. In addition, WS cells are hypersensitive to hydroxyurea, which stalls replication forks without inducing DNA adducts [32,39]. Because interstrand crosslinks and perturbed replication forks are believed to be repaired through HR, these results suggest an important role for WRN in recombinational resolution of stalled and collapsed replication forks.

Mounting in vitro evidence suggests that WRN acts on a plethora of DNA replication fork structures. For instance: a. WRN’s unwinding and pairing activities function in the regression of a replication fork substrate model [27,73]; b. WRN performs a strand-exchange function through its DNA unwinding activities [74]; c. WRN not only binds to D-loop structures, but also uses its helicase and exonuclease activities to disrupt and degrade the D-loops [75,76]; d. WRN acts on HJ substrate to convert it into a four-stranded replication fork structure, a key step of branch migration [77]; and e. WRN works on G-rich telomeric sequences to form t-loops [78]. Overall, these studies provide strong evidence that DNA substrates that mimic various HRR intermediate structures are suitable substrates for WRN’s nuclease activities, through which WRN contributes to the HRR pathway.

HRR not only requires the regulated activities of various factors, it also involves the post-translational modifications of these factors. WRN interacts with a range of key proteins of the HRR pathway. For example, WRN binds to the MRE11/RAD50/NBS1 (MRN) complex [24] and the recombination mediator, RAD52 [36]. WRN also interacts physically and functionally with RPA [79] and Breast cancer associated protein 1 (BRCA1 [80]. Furthermore, WRN co-localizes with RAD51, a key enzyme in HRR, at the sites of perturbed replication forks [81]. In addition to these protein-protein interactions, WRN is also post-translationally modified by ATM, ATR, c-Abl and CDK1 in response to replication stress (see Section 3 for details). These studies imply that WRN acts in concert with a variety of factors to facilitate HRR.

## 6. Consequences of WRN Deficiency and Replication Stress

Premature aging, cancer, and cardiomyopathy are the major symptoms exhibited by patients with WS. Most of the studies involving wild type WRN or WRN mutants harboring exonuclease, helicase, ATPase, post-translational modifications, or fragmented WRN concluded that WRN is important both for maintaining genome stability and for suppressing premature senescence. However, determining how cells decide whether to undergo premature senescence or cancer in the absence of WRN requires additional experimental evidence. Nonetheless, genome instability and premature senescence are the two major phenotypes that have been well studied in WRN-defective cells.

### 6.1. Role of WRN in Maintaining Genome Stability

Patients with WS have a high incidence of malignant neoplasms, and the tumor type of the neoplasms that appear in these patients differs from that observed in normal aging: in patients with WS, mesenchymal and epithelial cancers are equally common, even though epithelial cancers appear ten times more often than mesenchymal cancers in the normal aging population [82,83,84,85,86]. Furthermore, somatic cells derived from patients with WS are predisposed to various genetic mutations, including chromosomal translocations, inversions, and deletions; and WS fibroblasts transformed with simian virus 40 (SV40) showed a high rate of spontaneous mutations [4]. Improper DNA repair accounts for the cellular phenotypes often found in WS, such as variegated translocation mosaicism [87] and extensive deletions of endogenous genomic loci [8]. WRN can be viewed as a tumor-suppressor gene because of its involvement in genome stability maintenance functions.

According to a model proposed by Su et al. (2016), after genotoxic stress-induced replication stress, WRN is recruited to the collapsed replication forks by NBS1 [22] and phosphorylated at S1141 by ATR [48]. This phosphorylation facilitates WRN ubiquitination, which modulates its dynamic interaction with collapsed replication forks (probably by affecting the interaction between NBS1 and WRN). This, in turn, facilitates HR-mediated repair of replication-associated DSBs by granting access to factors (probably MUS81/EME1) involved in these processes. Finally, ubiquitinated WRN is targeted to the proteasome-dependent degradation pathway. These processes work together to maintain genomic stability under collapsed replication fork conditions. If WRN is not phosphorylated by ATR, WRN ubiquitination is reduced, resulting in a stable interaction between WRN and replication-associated DSBs, which prevents other factors involved in replication and repair from binding to those replication-associated DSBs. As a result, cells cannot resolve recombination intermediates that arise after RAD51-dependent strand invasion, eventually leading to anaphase bridge formation and chromosome instability.

The contribution of chromosome instability to the initiation of cancer in individuals with WS is still elusive. Though exposure to different replication stress inducers compromises WS cells’ survival, some cells with chromosomal aberrations enter mitosis. Every subsequent round of replication increases the overall mutation level in surviving cells. Thus, defective replication fork processes are biologically significant, because replicating a damaged genome provides opportunities for genomic rearrangements and can increase genomic instability, leading to the genetic changes that make an initiated cell progress to a malignant cell.

### 6.2. Role of WRN in Suppressing Premature Aging

Premature aging is the major phenotype of patients with WS [88]. We can distinguish two types of cellular senescence: replicative senescence, which depends on telomere shortening, and premature senescence induced by genotoxic stress, which occurs without telomere shortening. Though replicative senescence mediated by telomere shortening is very slow, genotoxic stress initiates rapid premature senescence. Telomere shortening, defective telomere maintenance and imperfect replication fork processing are the most studied causes of cellular senescence in WRN-defective cells [51,64]. Though the initial signal for cellular senescence can originate from various regions in the genome, evidence indicates that these genomic insults, irrespective of the genomic loci, proceed through the same DNA damage response signaling pathways. Specifically, double-stranded DNA ends revealed by telomere deprotection or generated by DNA damaging agents or replication stress initiate the ATM-dependent pathway; similarly, regions of single-stranded DNA at telomeres or at stalled replication forks initiate the ATR-dependent pathway. Both the ATM and ATR pathways can induce a senescence phenotype in response to an appropriate genomic lesion, whether WRN-deficiency is involved in producing that set of circumstances or not. Thus, several different genomic events can induce senescence in the absence of a functional WRN. However, it remains unclear how much telomere shortening, deprotected telomeres and replication stress induced by genotoxic agents in both telomeric and non-telomeric genomic loci each contribute to premature senescence in the absence of a functional WRN.

## 7. Conclusions and Future Perspectives

Though WRN’s nuclease and non-nuclease activities have been implicated in a multitude of DNA metabolic pathways, the mechanisms that regulate WRN activity to prevent carcinogenesis and premature aging at the nucleotide level have not been well established. Deciphering the molecular choreography of WRN and its biochemical activities, post-translational modifications, and interaction partners in promoting faithful replication fork processing will provide new insight into the molecular origin of cancer and aging phenotypes in individuals with WS. Identifying WRN phosphorylation events and interacting proteins will reveal novel mechanisms that explain how post-translational modifications induced by replication stress contribute to WRN’s biological functions. Decoding the mechanism that WRN uses to stabilize its interacting partners at replication-associated DSBs will further advance our understanding of the pathophysiology of aging.

How can we exploit what we know about WRN’s contributions to replication fork stability to treat cancer or prevent premature aging-associated phenotypes? 1. WRN phosphorylation redirects WRN to degrade in response to replication stress. Can this property of WRN be used to eliminate WRN-proficient cancer cells using combination therapy approaches? 2. Recently, a small molecule inhibitor that prevents WRN’s helicase activity was identified [89]. Since the helicase activity of WRN is critical for resolving perturbed replication forks, WRN helicase inhibitors in combination with cancer therapeutics that cause replication stress can be used to sensitize WRN-proficient cancer cells. However, it will be challenging to apply WRN’s known functions in replication to treat or prevent symptoms associated with premature aging due to WRN deficiency. On the brighter side, lessons learned from individuals with WS can be applied to treat diseases associated with normal biological aging.

While the accumulation of senescent cells and the induction of the senescence-associated secretory phenotype (SASP) could contribute to aging features in WS, we do not know what signaling events, beyond the activation of ATM and ATR checkpoint pathways, induce and maintain the senescence phenotype in the absence of a functional WRN. Evidence indicates that the accumulation of chromatin fragments, or micronuclei, in the cytoplasm due to genomic instability can initiate a cytosolic DNA sensing pathway-mediated premature senescence mechanism that does not involve telomere shortening [90,91,92,93,94]. A recent study showed that cytosolic DNA fragments released in response to replication stress and DNA damage are sensed by the cyclic GMP-AMP synthase (cGAS)-stimulator of interferon genes (STING)-mediated cytosolic DNA sensing pathway, which activates both nuclear factor kappa-light-chain-enhancer of B-cells (NF-κB) and signal transducer and activator of transcription 1 (STAT1)-mediated immune signaling and cellular senescence [95]. Similarly, Bhattacharya et al. (2017) found that lack of RAD51 results in excessive processing of newly replicated genomic DNA by MRE11, and the resulting genomic DNA fragments accumulate in the cytosol, ultimately activating inflammatory signaling [96]. In the absence of WRN, MRE11’s exonuclease activity also acts on the newly replicated genomic DNA in response to replication stress; however, whether these degraded DNA trigger immune signaling that contributes to the premature senescence phenotype in WS cells remains to be investigated [22]. It is worth noting that fibroblasts derived from patients with WS and helicase-mutant mice and serum from patients with WS show heightened inflammatory signaling [97,98,99]. However, further experiments are required to determine whether the cytosolic DNA sensing pathway plays a role in initiating immune signaling-driven premature senescence in patients with WS.

Cellular senescence restricts unlimited cellular proliferation and plays critical roles in both aging and tumor suppression. However, it is intriguing that individuals with WS exhibit the symptoms of both. Several studies show that senescent cells develop SASP that may induce changes in the tissue microenvironment, causing it to gain control over cell behavior and promoting tumorigenesis. However, future experiments are required to show that immune signaling initiated by genomic instability is the primary trigger for the development of SASP-mediated cancer in patients with WS.
